# Structural Degradation in Midcingulate Cortex Is Associated with Pathological Aggression in Mice

**DOI:** 10.3390/brainsci11070868

**Published:** 2021-06-29

**Authors:** Sabrina van Heukelum, Femke E. Geers, Kerli Tulva, Sanne van Dulm, Christian F. Beckmann, Jan K. Buitelaar, Jeffrey C. Glennon, Brent A. Vogt, Martha N. Havenith, Arthur S. C. França

**Affiliations:** 1 Donders Institute for Brain, Cognition and Behavior, Radboudumc, 6525 EN Nijmegen, The Netherlands; femkegeers@hotmail.com (F.E.G.); kerli.tulva@hotmail.com (K.T.); sanne.vandulm@student.hu.nl (S.v.D.); c.beckmann@donders.ru.nl (C.F.B.); Jan.Buitelaar@radboudumc.nl (J.K.B.); jeffrey.glennon@ucd.ie (J.C.G.); martha.havenith@esi-frankfurt.de (M.N.H.); arthursergiof@gmail.com (A.S.C.F.); 2 Department of Cognitive Neuroscience, Radboudumc, 6525 EN Nijmegen, The Netherlands; 3 Conway Institute of Biomolecular and Biomedical Research, School of Medicine, University College Dublin, Belfield, D04 Dublin, Ireland; 4 Cingulum Neurosciences Institute, Manlius, NY 13104, USA; bvogt@twcny.rr.com; 5 Department of Anatomy and Neurobiology, Boston University School of Medicine, Boston, MA 02118, USA; 6 Zero-Noise Lab, Ernst Strüngmann Institute for Neuroscience, 60528 Frankfurt am Main, Germany

**Keywords:** cingulate cortex, aggression, neuronal degeneration, astrogliosis, cFos, resident-intruder test

## Abstract

Pathological aggression is a debilitating feature of many neuropsychiatric disorders, and cingulate cortex is one of the brain areas centrally implicated in its control. Here we explore the specific role of midcingulate cortex (MCC) in the development of pathological aggression. To this end, we investigated the structural and functional degeneration of MCC in the BALB/cJ strain, a mouse model for pathological aggression. Compared to control animals from the BALB/cByJ strain, BALB/cJ mice expressed consistently heightened levels of aggression, as assessed by the resident-intruder test. At the same time, immunohistochemistry demonstrated stark structural degradation in the MCC of aggressive BALB/cJ mice: Decreased neuron density and widespread neuron death were accompanied by increased microglia and astroglia concentrations and reactive astrogliosis. cFos staining indicated that this degradation had functional consequences: MCC activity did not differ between BALB/cJ and BALB/cByJ mice at baseline, but unlike BALB/cByJ mice, BALB/cJ mice failed to activate MCC during resident-intruder encounters. This suggests that structural and functional impairments of MCC, triggered by neuronal degeneration, may be one of the drivers of pathological aggression in mice, highlighting MCC as a potential key area for pathologies of aggression in humans.

## 1. Introduction

Aggression is a fundamental feature of the behavioral repertoire across species, ranging from fish to rodents, primates and humans [1,2]. While it can clearly serve an adaptive role, e.g., in situations that require protecting one’s own or one’s offspring’s survival, aggression can quickly lose its adaptive benefits if used out of context or proportion [3]. Such maladaptive aggressive behavior is observed as a symptom in a multitude of psychiatric conditions, including psychopathy, anti-social or borderline personality disorder, and even Alzheimer’s disease [4,5,6,7]. All of these disorders have something else in common: an involvement of cingulate cortex [4,8,9,10].

The overall importance of cingulate cortex for the control of aggression has been confirmed repeatedly [1,11,12,13,14,15], and there is evidence that its sub-regions—particularly anterior cingulate cortex (ACC) and midcingulate cortex (MCC)—work in tandem to regulate aggressive behavior. For instance, a mouse model of pathological aggression, the BALB/cJ mouse, shows increased ACC volumes but decreased MCC volumes [16] as well as differential changes in the distribution of parvalbumin- and somatostatin-expressing interneurons across ACC and MCC [17]. Similarly, patients with aggressive disorders display hypoactivation of MCC [4,11] as well as ACC [4,18]. In addition, the border areas joining MCC and ACC show volumetric reductions in patients with anti-social personality disorder [19]. However, the specific contributions of ACC and MCC to this process are less well-defined [20,21,22,23,24]. Recent work has begun to elucidate ACC’s function, highlighting its role in curbing pathological aggression in human patients and animal models [4,7,25,26,27]. In contrast, the function of MCC has not been clearly delineated yet. Human neuroimaging data suggest MCC as a hub in a network associated with the cognitive modulation of aggressive behavior [11]. However, since rodent MCC has so far rarely been studied in isolation from ACC [16,17,20,21,23,24], there are few mechanistic insights into MCC’s specific role in the control of aggressive behavior.

Here, we demonstrate new links between the structural and functional degradation of MCC and the occurrence of pathological aggression in BALB/cJ mice. Specifically, we show that MCC in BALB/cJ mice sustains high rates of neuron death and associated decreases in neuron density as well as reactive astrogliosis. As a consequence, BALB/cJ mice fail to activate MCC during aggressive encounters, as shown by subsequent cFos staining. These results suggest that in the BALB/cJ strain, structural degeneration of MCC prevents it from being recruited during confrontations. This is in line with studies showing decreased MCC activity in aggressive patients, and further highlights structural degeneration across cingulate cortex as a potential root cause of pathological aggression.

## 2. Materials and Methods

### 2.1. Animals and Housing Conditions 

BALB/cJ (total *N* = 14) and BALB/cByJ (total *N* = 13) mice were obtained from the Jackson Laboratory (Bar Harbor, ME, USA); C57BL/6J intruder mice were obtained from Charles River Laboratories (Erkrath, Germany). At all times, mice had free access to food and water and were housed in an enriched environment (High Makrolon^®^ cages with Enviro Dri^®^ bedding material and Mouse Igloo^®^). To be able to test the animals during their active phase, they were exposed to a reversed 12–12 h day–night cycle with sunrise at 7.30 pm. Resident mice (BALB/cJ and BALB/cByJ) were housed individually, while intruder mice were housed in groups of 5–6 animals per cage. At the start of the RI test, all resident mice were 11 weeks old and all intruder C57BL/6J mice were 7 weeks old. Animal procedures were conducted in compliance with EU and national regulations as well as local animal use ethical committees (European Directive 2010/63/EU), and approved by the Ethics Committee on Animal Experimentation of Radboud University (RU-DEC number 2017-0032). From both BALB strains, 6 animals were not exposed to the RI test to exclude the possibility that the RI test might have an effect on structural markers.

### 2.2. RI Test

Resident mice were isolated and housed individually 10 days prior to testing. The testing was performed in the home cage of the BALB/cJ and BALB/cByJ mice in a dark room with red overhead lighting. An infrared camera (SuperLoLux, JVC) was used to videotape the behavior. Testing was done for five consecutive days, and each day, each BALB/cJ and BALB/cByJ mouse was confronted with a different C57BL/6J intruder mouse. The order of testing was randomized and the first test was started 1 h after the beginning of the dark phase. At the beginning of the test, an intruder animal was placed in the home cage of the resident animal, separated by a glass screen to allow for visual and olfactory stimulation for 5 min. After this instigation phase, the glass screen was removed and interaction was allowed for 5 min. All recordings were scored by the same researcher who was blind to the strain of the animal (BALB/cJ and BALB/cByJ mice have the same appearance). Manual scoring consisted of determining attack latency, threatening behavior (tail rattles) and bites (including bite distribution), using the program The Observer (Noldus). An attack was defined as a bite directed at the back, tail, abdomen, neck or face of an intruder [16,28]. For analyses, we further separated bites directed to robust body areas, specifically the back (species-typical) from attacks directed to the belly, flank, neck or face. These latter are considered as species-atypical bites as they have the potential to inflict lasting, and potentially lethal harm on the intruder, and are therefore not part of the normal behavioral repertoire for territorial confrontations between mice [16,17]. The individual aggressive behaviors were then averaged across all days and then analyzed with a one-way ANOVA (strain as between-subject factor). All statistical analyses were carried out using SPSS23-software (SPSS Inc., Chicago, IL, USA). The false discovery rate method [29] was used to correct for multiple comparisons for all behavioral data. Note that the behavioral results shown here partly stem from a cohort that was previously also studied in [25], but here, we relate them to new structural and functional measurements of MCC.

### 2.3. Perfusion and Tissue Preparation

45 to 55 min after the last RI test, all BALB/cJ and BALB/cByJ mice were deeply anesthetized with isoflurane (3–5%) and perfused with saline, followed by 150 mL of 4% paraformaldehyde solution (PFA) in 0.1 M phosphate buffer (PBS). Brains were removed, fixed overnight in 4% PFA and then kept in 0.1 M PBS at 4-degree celsius temperature. One day before cutting, brains were placed in 0.1 M PBS plus 30% sucrose to ensure cryoprotection. Coronal sections (30 μm) were obtained on a freezing microtome (Microm, Thermo Scientific, Waltham, MA, USA). All sections containing MCC were placed in running order in containers filled with 0.1 M PBS + 0.01% sodium azide (to prevent fungal contamination) and stored at 4-degree temperature until use.

### 2.4. Cell Death and Neuronal Density across MCC Layers

As done in [16], we mounted all slices containing A24’ (MCC) on gelatine-coated slides (0.5% gelatine + 0.05% potassium chromium (III) sulphate). Slices were then air-dried and placed in a stove at 37 °C overnight. The next day, sections were first placed in a 96% alcohol bath for 10 min, then hydrated in graded alcohol baths (1 × 70%, 1 × 50%, 2 min each), dehydrated in graded alcohol baths (1 × 70%, 1 × 96%, 1 × 100%, 2 min each) and stained in a 0.1% cresyl violet solution for approximately 5 min. Afterwards, sections were placed in a graded alcohol series (3 × 95%, 3 × 100%, 2 min each), cleared in xylene (Sigma–Aldrich, St. Louis, MO, USA) and mounted with entellan (Sigma–Aldrich, St. Louis, MO, USA). One image of each section was then obtained on an Axioskop fs microscope using Neurolucida software (MBF Bioscience, Williston, VT, USA). To localize A24’, we used the Paxinos and Franklin mouse atlas [30] and relied on the same methodology as [16] to define the start/end of A24’. From each mouse, the MCC section was chosen at approximately the same anatomical level (at AP −0.655). First, the area of each layer was delineated and then the neurons in each layer counted. Counting was done using the optical fractionator tool in the program StereoInvestigator (MBF Biosciences, Williston, VT, USA), as this represents an unbiased method for cell counting. Each slice had the same counting frame (50 × 50) and grid size (100 × 100). Counting was done at a 60× magnification. The *estimated population using number weighted section thickness* value given by the program was used as value for neuronal density. Cells with a distinct nucleolus with or without several heterochromatin granules and a rim of cytoplasm around the nucleus were considered neurons. Those without a distinct nucleolus were not considered neurons. Next to those neurons, we counted neurons showing signs of cell death. These were defined as cells with an irregular, shrunken and/or crenulated shape and nuclear shrinkage as well as increased vacuolation and tissue disruption. The number of neurons was then calculated as the sum of healthy and pyknotic neurons per mm^2^ (dividing the value provided by the program for each individual layer by the surface area of that layer). To determine the proportion of healthy neurons vs. pyknotic neurons, we divided the number of pyknotic neurons by the number of healthy neurons. Next to the animals that performed the RI test, we included 6 BALB/cJ mice that had not been exposed to the test to exclude any effects of the RI test on neuronal degeneration. Data was analyzed with repeated measures ANOVAs (layer as within subject factor, strain as between subject factor) and *t*-tests were used as post hoc tests. The false discovery rate method [29] was used to correct for multiple comparisons.

### 2.5. Microglia and Astroglia across MCC Layers

To assess the number of microglia and astroglia, we used MCC sections of 15 BALB/cJ and 13 BALB/cByJ mice. For both strains, we included animals (*N* = 6 per strain) that did not perform the RI test. MCC sections were stained with a standard free-floating immunofluorescence protocol with antibodies for microglial and astroglial markers (Iba1 and GFAP and S100B, respectively). Sections were incubated overnight at room temperature in Iba1 anti-rabbit (1:1500, Wako, Osaka, Japan, product code: 019-19741) and GFAP anti-guinea pig (1:1500, Synaptic Systems, Goettingen, Germany, product code: 173004) as well as a marker for neurons (NeuN anti-chicken, 1:1000, Millipore, Burlington, MA, USA, product code: ABN91). The next day, sections were incubated in matching secondary antibodies at room temperature (Alexa Fluor donkey anti-rabbit 488, Abcam, Cambridge, MA, USA, product code: ab150061), Alexa Fluor donkey anti-guinea pig 647 (Jackson Immuno Research Europe Ltd., Ely, Cambridgeshire, UK, product code: 706-605-148) and Alexa Fluor goat anti-chicken 555 (Thermo Fisher Scientific, Waltham, MA, USA, product code: A-21437)). Photographs of the MCC sections were then taken with an Axio Imager.A2 microscope (Zeiss, Koeln, Germany) at 20× magnification and analyzed with Fiji (ImageJ). We used the NeuN stain to outline the different layers, saved these as ROIs and then applied them to the Iba1 and GFAP stains. Microglia and astroglia were then manually counted and their number divided by the surface area of the layer to attain the number of glia per mm^2^. Given that GFAP in gray matter is known to often preferentially stain reactive astroglia [31], we decided to stain for another astroglia marker, S100B, in combination with a marker for reactive toxic astroglia (Serping1). This enabled us to determine the total number of astroglia regardless of their activity state as well as the percentage of toxic and neuroprotective reactive astroglia. As done previously, we used a standard free-floating immunofluorescence protocol and incubated the MCC sections overnight in S100B anti-guinea pig (1:1000, Synaptic Systems, Goettingen, Germany, product code: 287004) together with Serping1 anti-mouse (Santa Cruz, Dallas, TX, USA, product code: sc-377062) as a marker for neurotoxic A1 astroglia. The next day, sections were incubated with matching secondary antibodies (anti-rabbit and anti-guinea pig were the same ones as previously used as well as an Alexa Fluor goat anti-mouse 555 (Abcam, Cambridge, MA, USA, product code: ab150114)) and a DAPI stain was added. Photographs of the MCC sections were then taken with an Axio Imager.A2 microscope (Zeiss, Koeln, Germany) at 20× magnification and analyzed with Fiji (ImageJ). Layers were outlined with the DAPI stain, ROIs saved and applied to the S100B and Serping1 stains. Positively stained cells were then manually counted and Serping1 markers were overlayed with the S100B markers to count the number of double-stained cells. The number of S100B positive cells was divided by the surface area of the specific layer and the number of A1 astroglia was determined by calculating the percentage of S100B + Serping1 double-stained sections. We then analyzed the data with repeated ANOVAs (layer as within-subject factor, strain as between subject factor) and *t*-tests were used as post hoc tests. The false discovery rate method [29] was used to correct for multiple comparisons.

### 2.6. cFos Staining for Functional Activation of MCC

To quantify MCC activation, we used brain sections of 15 BALB/cJ and 13 BALB/cByJ mice. As previously, for both strains, we included animals (*N* = 6 per strain) that did not perform the RI test to check for baseline activity of MCC. Mice subjected to the RI test were then sacrificed 45–55 min after the final RI test (see ‘Behavior’ section of Materials and Methods). MCC sections were stained with a standard free-floating immunofluorescence protocol with an antibody for cFos. Briefly, sections were incubated overnight at room temperature in cFos anti-guinea pig (1:1000, Synaptic Systems, Goettingen, Germany, product code: 226004). The following day, the sections were incubated in a matching secondary antibody at room temperature (Alexa Fluor donkey anti-guinea pig 647) and a DAPI stain was added. Photographs of the MCC sections were then taken with an Axio Imager.A2 microscope (Zeiss, Koeln, Germany) at 20× magnification and analyzed with Fiji (ImageJ). Layers were outlined with the DAPI stain, ROIs saved and applied to the cFos stain. Positively stained cells were then manually counted. The number of cFos positive cells was divided by the surface area of the specific layer. We then analyzed the data with repeated ANOVAs (layer as within subject factor, strain as between subject factor) and *t*-tests were used as post hoc tests. The false discovery rate method [29] was used to correct for multiple comparisons.

## 3. Results

### 3.1. BALB/cJ Mice Are More Aggressive Than BALB/cByJ Mice

**Figure 1:**
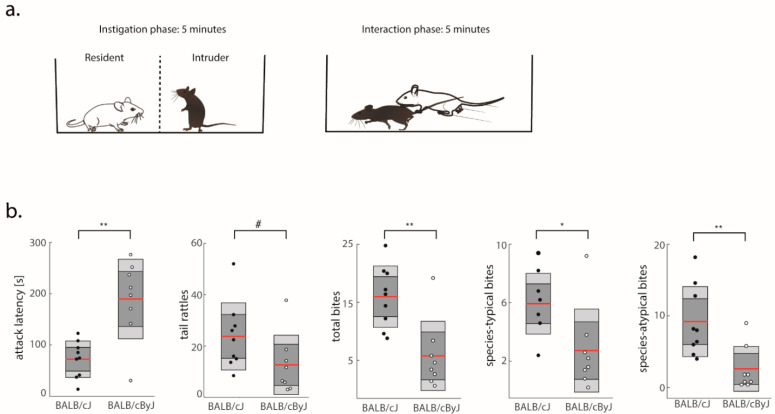
Behavioral metrics of aggression: (**a**) Schematic of the RI test. Testing started with a 5 min instigation phase (mice separated by a glass screen), followed by a 5 min interaction phase; (**b**) Average attack latency, total number of bites, tail rattles, species-typical bites, and species-atypical bites. Black dots, BALB/cJ mice; gray dots, BALB/cByJ mice. Shown are average (red line), 95% confidence interval (dark gray area), and 1 SD (light gray area). ^#^ *p* < 0.1, * *p* < 0.05; ** *p* < 0.01.

In line with previous studies [16,17], BALB/cJ mice showed more aggression than BALB/cByJ mice across all behavioral measurements derived from the RI test (Figure 1a). On average, BALB/cJ mice attacked faster than BALB/cByJ mice (*F* (1, 15) = 16.75, *p* = 0.005, *η*^2^ = 0.53; Figure 1b), issued more threats (tail rattles, (*F* (1, 15) = 3.4, *p* = 0.085, *η*^2^ = 0.19), and engaged in biting behavior more often than BALB/cByJ mice (*F* (1, 15) = 14.01, *p* = 0.005, *η*^2^ = 0.48; Figure 1b). To further dissect the behavioral profile of BALB/cJ mice, we separately scored the occurrence of bites to different body parts. Specifically, bites to the back typically do not result in serious harm to the opponent and tend to be part of the species-typical repertoire of aggressive acts in territorial disputes [16,25]. In contrast, bites directed at vulnerable body parts like the abdomen and neck are potentially more harmful and rarely occur in territorial disputes between wild-type mice [32,33]. We therefore consider such bites as species-atypical or pathological. While BALB/cJ mice did inflict back bites more frequently than BALB/cByJ mice (*F* (1, 15) = 7.2, *p* = 0.02, *η*^2^ = 0.32), the most dramatic increase was observed in the frequency of species-atypical bites (*F* (1, 15) = 10.62, *p* = 0.008, *η*^2^ = 0.42; Figure 1b). This shows that pathological forms of aggression are particularly elevated in the BALB/cJ strain.

### 3.2. Structural Degradation in MCC of BALB/cJ Mice

**Figure 2:**
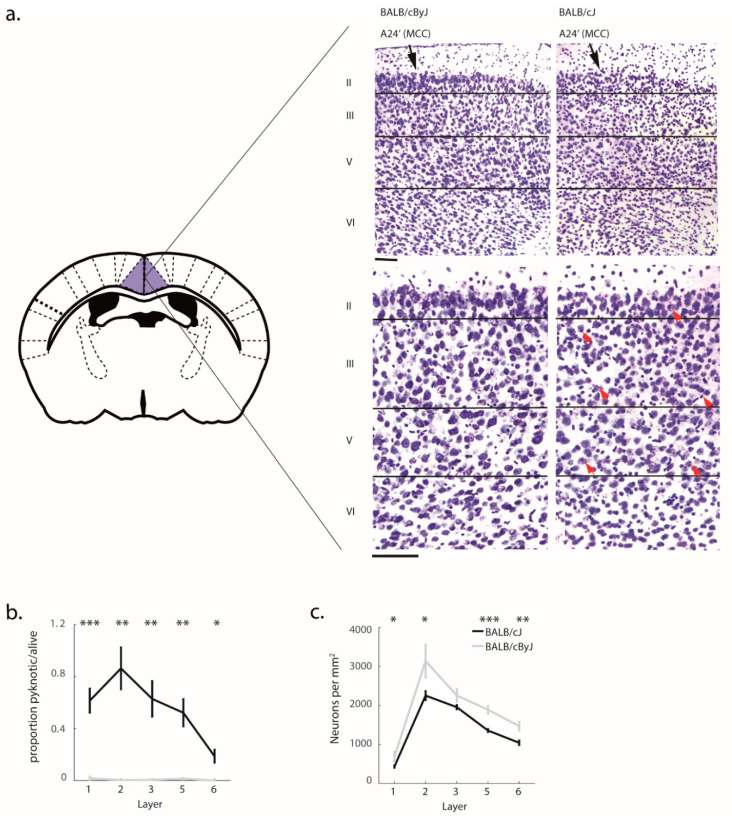
MCC histology: (**a**) Schematic of area MCC. Inset: photographs in different magnifications show the outlines of the position of layers. Pyknotic neurons are noted with red arrows in the high-magnification BALB/cJ case. There are also 2 black arrows in layer II to emphasize places where there are clearings with no neurons to emphasize their loss. Scale bars, 100 μm; (**b**) The proportion of pyknotic/non-pyknotic neurons. Black line, BALB/cJ mice; gray line, BALB/cByJ mice. Shown are average and SEM per layer; (**c**) The number of neurons per mm^2^. Shown are average and SEM per layer. * *p* < 0.05; ** *p* < 0.01; *** *p* < 0.001.

**Figure 3:**
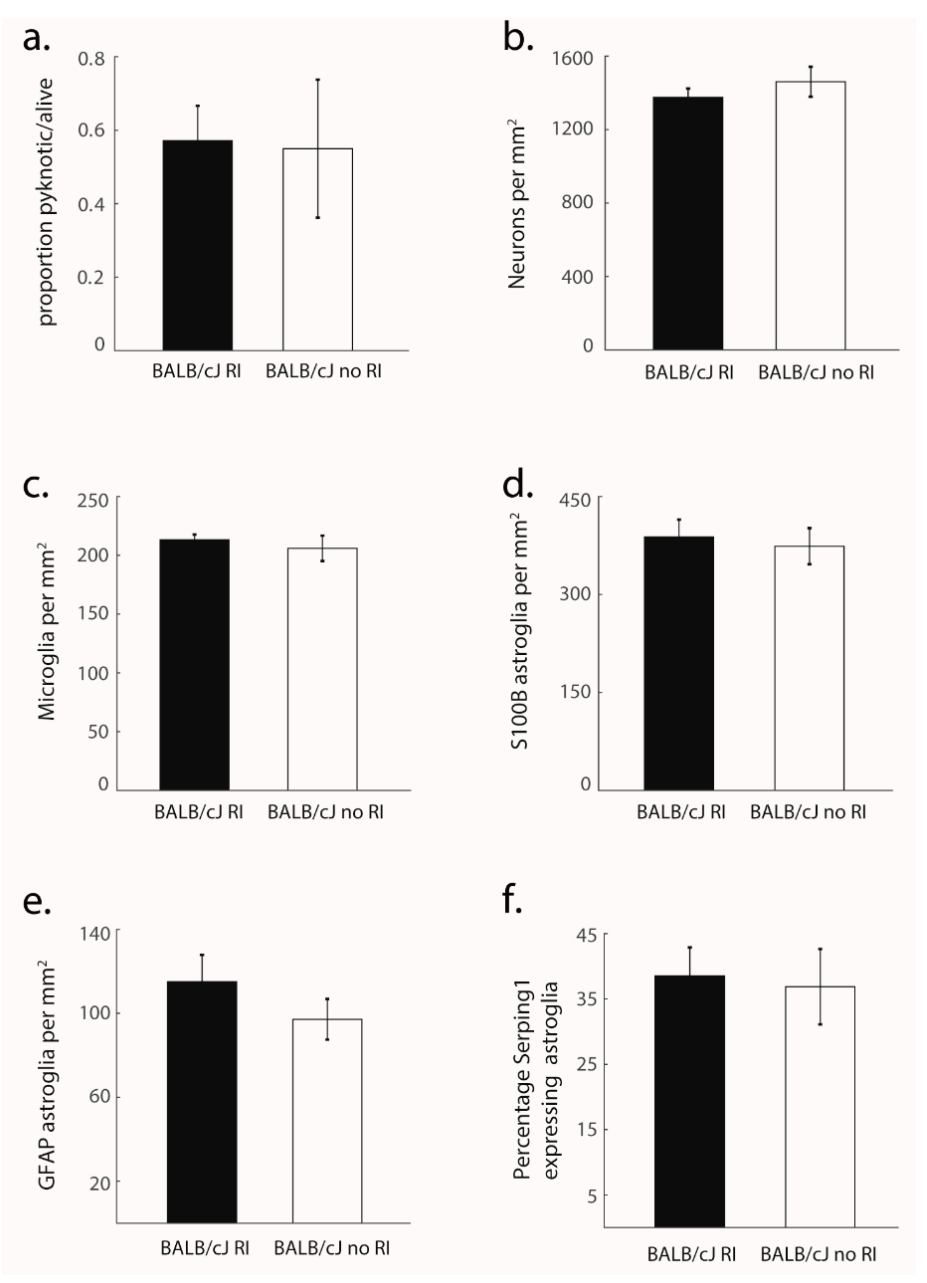
Histological measures in BALB/cJ animals with and without RI test: (**a**) Proportion pyknotic neurons/healthy neurons in MCC of BALB/cJ mice that participated in the RI (*N* = 9) test versus those that did not (*N* = 6). Shown are average and SEM; (**b**) Same as *a* for neuron density per mm^2^; (**c**) Same as *a* for microglia per mm^2^; (**d**) Same as *a* for S100B positive astroglia per mm^2^; (**e**) Same as *a* for GFAP positive astroglia per mm^2^; (**f**) Same as *a* for percentage toxic astroglia. None of the comparisons are significant.

Unlike BALB/cByJ mice, BALB/cJ mice exhibited advanced structural degradation of MCC (Figure 2). This included strikingly high proportions of pyknosis (*F* (1, 21) = 19.76, *p* < 0.001, *η*^2^ = 0.49) compared to virtually non-existent pyknosis in control mice, an effect that was evident across all cortical layers (all *p* < 0.01; Figure 2b). This degeneration seemed to already have affected neuronal density, as we observed significantly decreased neuronal density in BALB/cJ mice (*F* (1, 31) = 12.85, *p* = 0.005, *η*^2^ = 0.38; Figure 2c), spanning all layers (*p* < 0.05) except layer 3. Importantly, BALB/cJ mice tested in the RI test did not differ from BALB/cJ mice which did not experience the RI test (Figure 3), demonstrating that pyknosis and decreased neuron density were present irrespective of exposure to the RI test, and may be one of the factors responsible for the observed behavioral outcomes.

### 3.3. Glial Processes Are in Line with Neuron Death

**Figure 4:**
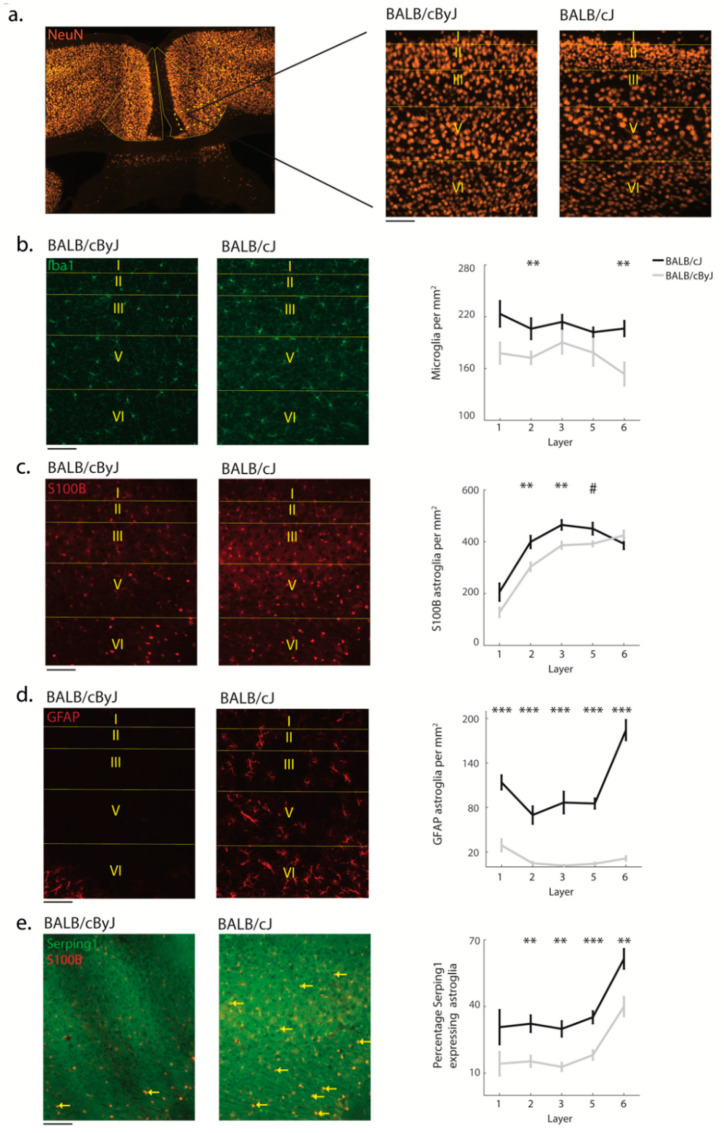
Microglia and astroglia across MCC layers: (**a**) Example photograph of a NeuN stained section at low magnification; rectangle shows the area of the zoom-in. Zoom-in: High-magnification photograph of a NeuN section with layers delineated. Scale bar: 100 μm; (**b**) Left and center panel: Example photograph of microglia across layers (left: BALB/cByJ, center: BALB/cJ); Right panel: Number of microglia per mm^2^ across layers. Black line, BALB/cJ; gray line, BALB/cByJ; (**c**) Same as (**b**) for S100B-positive stained astroglia across layers; (**d**) Same as (**b**) for GFAP-positive stained astroglia; (**e**) Left and center panel: Example photographs of S100B astroglia double stained with a marker for toxic astroglia (Serping1, yellow arrow); Right: The percentage of toxic astroglia. All right-hand panels show average and SEM. ^#^ *p* < 0.1, ** *p* < 0.01; *** *p* < 0.001.

In line with the fact that both microglia and astroglia are activated upon neuronal insult, we observed increases in the density of microglia (*F* (1, 26) = 14.1, *p* = 0.003, *η*^2^ = 0.35; Figure 4b) and astroglia (*F* (1, 27) = 6.95, *p* = 0.02, *η*^2^ = 0.21; Figure 4c) in MCC. Post hoc tests revealed that these differences were layer-specific: For microglia, differences were restricted to layers 2 (*p* < 0.01) and 6 (*p* < 0.01) and for astroglia, differences were seen in layers 2 (*p* < 0.01) and 3 (*p* < 0.01). To further determine the activity state of the proliferated glial populations, we tested for reactive astrogliosis. The number of reactive astroglia in BALB/cJ mice was dramatically increased across all layers (*F* (1, 26) = 104.5, *p* < 0.001, *η*^2^ = 0.8); with up to a ten-fold difference in some layers (Figure 4d). To decipher whether these reactive astroglia were toxic A1 astroglia, we stained for the Serping1 marker [34]. This analysis revealed strongly elevated levels of neurotoxic A1 astroglia in BALB/cJ mice compared to BALB/cByJ mice (*F* (1, 27) = 19.94, *p* < 0.001, *η*^2^ = 0.43; Figure 4e) located across all layers (all *p* < 0.01) except for layer 1. Again, there were no significant differences between mice that had performed the RI test and those that did not (Figure 3c–f). Consistently with previous findings regarding ACC [25], these results suggest a scenario in which neuronal insult triggers reactive astrogliosis including neurotoxic A1 astroglia, which then induce cell death in the affected neurons [35].

### 3.4. Functional Implications of Structural Degradation

**Figure 5:**
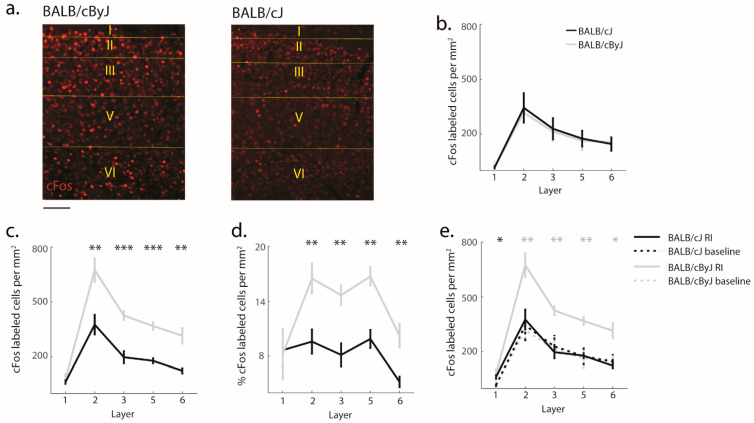
cFos activity levels: (**a**) Example photographs of stained cFos cells in BALB/cByJ and BALB/cJ mice, scale bar: 100 μm; (**b**) The number of cFos-labeled cells per mm^2^ at baseline. Black line, BALB/cJ mice; gray line, BALB/cByJ mice; (**c**) The number of cFos-labeled cells per mm^2^ during the last day of the RI test. Asterisks: Statistical significance of comparison between BALB/cByJ and BALB/cJ mice; (**d**) Same as (**b**) but expressed as percentage of total neuron density; (**e**) Comparison of activity at baseline versus RI for BALB/cJ and BALB/cByJ mice, respectively. Gray asterisks: Statistical comparison between baseline and RI test in BALB/cByJ animals. Black asterisk: Same for BALB/cJ; (**b**–**e**) Show the average and SEM per layer. * *p* < 0.05; ** *p* < 0.01; *** *p* < 0.001.

The dramatic structural change of MCC we observed raises the question whether the functional activation of MCC was also impacted. To address this question, we used cFos staining in order to track activity levels in MCC at baseline (i.e., before animals had ever encountered the RI test) and triggered by the RI test (Figure 5a). We found no differences in MCC activity between BALB/cJ and BALB/cByJ mice at baseline (*F* (1, 9) = 0.02, *p* = 0.92, *η*^2^ = 0.002; Figure 5b). In contrast, MCC activity in response to aggressive encounters was reduced in BALB/cJ mice compared to BALB/cByJ mice (*F* (1, 15) = 22.63, *p* < 0.001, *η*^2^ = 0.60). This difference held even when we controlled for differences in neuron density (Figure 5c,d). On average, BALB/cByJ mice activated more than twice the number of MCC neurons during the RI test than BALB/cJ mice (Figure 5). This effect was seen across all cortical layers (all *p* < 0.001) except for layer 1. Consistently with this, there was no significant change between MCC activity for the baseline and RI conditions in BALB/cJ mice, while there was a significant increase of MCC activity in BALB/cByJ mice (BALB/cJ: *F* (1, 13) = 0.013, *p* = 0.92, *η*^2^ = 0.001; BALB/cByJ: *F* (1, 11) = 18.37, *p* = 0.003, *η*^2^ = 0.63; Figure 5e). This suggests that while baseline activity in MCC was unaffected by the structural degradation we observed, the ability to upregulate MCC activity in response to confrontational interactions was lost.

## 4. Discussion

Here we demonstrate a structural and functional breakdown of MCC in a mouse model of pathological aggression, the BALB/cJ mouse. We first show that BALB/cJ mice behave more aggressively than control mice, in terms of species-typical tail rattles and back bites as well as species-atypical bites to vulnerable body parts as reported previously [16,17,25]. On a structural level, we then demonstrate a strongly elevated rate of neuron death and decreased neuron density across MCC, together with increases in the number of microglia and astroglia, reactive astrogliosis and an upregulation of neurotoxic astroglia. These structural changes were accompanied by functional impairments. Unlike control mice, BALB/cJ mice failed to activate MCC during aggressive encounters. This suggests MCC pathology as a significant contributor to behavioral deficits in BALB/cj animals.

A question regarding these results is whether BALB/cByJ animals represent an appropriate control strain for BALB/cJ animals. The advantage of this approach [28,36] is that BALB/cJ and BALB/cByJ animals are genetically very similar [28], but differ strongly in their aggression levels [16,17,28,37]. It is, however, important to note that BALB/cByJ animals are not a ‘neutral’ control strain, if such a thing exists [38]—for instance, they are more anxious and less social than wild-type animals [39,40]. This would critically impact our conclusions if BALB/cByJ animals also showed altered aggressive behavior, e.g., by acting more tamely. This does not seem to be the case: BALB/cByJ animals have been reported to act slightly more aggressively than wild-type animals when housed in large groups [41].

One of the most striking findings of this study is the pervasive neuron death occurring across MCC, accompanied by reactive astrogliosis. While we cannot prove conclusively how these degenerative processes are triggered, insights from a previous study demonstrating similar structural degeneration in ACC [25] suggest that neuron death occurs first, gradually spreading outwards from cingulate cortex and triggering reactive astrogliosis in its wake. This scenario was supported by the observation that laterally neighboring areas of ACC, specifically M2, S1 and insula, were decreasingly affected by neuronal degeneration with distance to ACC, and did not show diminished neuron density [25]. In addition, we also checked for glial changes in M2, which borders directly onto ACC, and did not observe any toxic astrogliosis there—signaling that while neuronal degeneration had spread beyond the borders of cingulate cortex, neither neuron density loss nor astrogliosis had. In the current study, MCC in animals of the same age was affected by neuronal degeneration as well as decreased neuron density and astrogliosis, suggesting that together with ACC, MCC is part of the ‘epicenter’ of a structural degeneration that progressively spreads across cortical areas in BALB/cJ animals. If this interpretation is correct, then with progressing age, BALB/cJ animals should experience an increasing spread of neuronal degeneration across cortical areas, with astrogliosis and loss of neuron density following at a delay. Future studies conducting structural analyses across brain areas and across the life span of BALB/cJ mice would be highly valuable in elucidating this question further.

cFos staining directly following the RI test indicated that these structural changes reduced MCC activity during confrontational encounters. This finding is consistent with the idea that failure to activate MCC during confrontations leads to the excessive aggression observed in BALB/cJ animals [16,17,25,28,37]. To support such a scenario more conclusively, future studies would need to add several pieces of information. First, manipulation of MCC activity in BALB/cJ mice could demonstrate to what extent activity levels in MCC directly modulate aggressive behavior on a moment-by-moment basis [25,26,27,42]. Second, given that ACC activity acts as a powerful ‘control dial’ for pathological aggression in BALB/cJ mice [25], the question arises whether MCC makes a unique contribution to the control of aggression, whether ACC and MCC play redundant or complementary roles, or whether ACC degradation is the main driver of pathological aggression in BALB/cj mice, with MCC degradation just occurring as a side effect. To answer these questions will require extensive follow-on work recording and manipulating neuronal populations in ACC and MCC individually and jointly in order to dissect the contributions of each area.

However, based on current knowledge, we can make some predictions. First, since rodent ACC and MCC are strongly interconnected, they are likely to act in concert at least to some extent, and unlikely to fulfil entirely separate functions. Second, given their divergent connectivity to other areas [20,23,24], ACC and MCC seem set up to enact complementary functions. Human MCC’s connectivity profile features connections to regions such as dorsolateral prefrontal cortex as well as insula and motor cortices, and, as such, seems ideally positioned to integrate internal models of the environment to guide decision-making and initiate action [20,21,22,43,44,45,46]. Consistently with this, a recent meta-analysis of functional brain alterations in aggressive individuals highlighted a network consisting of anterior MCC, right rolandic operculum, precentral gyrus and precuneus cortex [11]. Within this network, MCC appeared to act as a central hub, assembling internal (pain, negative affect) and external information (environmental cues) to inform context-appropriate motor responses [11,22,47,48]. In rodents, MCC additionally receives strong connections from sensory integration areas such as retrosplenial cortex [23,24]. Impairments of MCC might therefore impact the animal’s ability to correctly process and respond to environmental stimuli, e.g., recognising submissive behavior as an incentive to cease aggressive actions.

In contrast, ACC is more widely connected to subcortical structures such as amygdala and hypothalamus, as well as autonomic brainstem nuclei [20,23,24]. This distribution suggests a division of labor whereby MCC drives decision making based on internal and external information, which is then enacted by ACC’s efferents to amygdala, hypothalamus and autonomic brainstem nuclei. This idea of complementary processing is also consistent with previous work demonstrating coordinated structural changes across cingulate cortex of aggressive BALB/cJ animals, for instance, increased ACC but decreased MCC volumes [16] and selective decreases of parvalbumin-positive (PV) interneurons in MCC but not ACC [17].

Most importantly, our results illustrate the importance of studying rodent MCC independently of ACC in order to define its function more clearly. Mainly due to conflicting nomenclatures [20,23,24,49], this has rarely been undertaken so far. As a result, the unique contribution of MCC to behavior, particularly in rodents, is yet to be explored, in terms of structure and function as well as in health and disease. The present study provides first insights into the crucial involvement of rodent MCC in the adaptive control of aggression.

## Data Availability

All data are available upon request to the corresponding author.
